# The Role of Peripheral CNS-Directed Antibodies in Promoting Inflammatory CNS Demyelination

**DOI:** 10.3390/brainsci7070070

**Published:** 2017-06-22

**Authors:** Silke Kinzel, Martin S. Weber

**Affiliations:** 1 Institute of Neuropathology, University Medical Center, 37099 Göttingen, Germany; silke.kinzel@med.uni-goettingen.de; 2 Institute of Neuropathology and Department of Neurology, University Medical Center, Georg August University, Robert-Koch-Str. 40, 37099 Göttingen, Germany

**Keywords:** multiple sclerosis, neuromyelitis optica, aquaporin-4, myelin oligodendrocyte glycoprotein, opsonization, autoantibody, central nervous system, CNS-draining lymphatics

## Abstract

In central nervous system (CNS) demyelinating disorders, such as multiple sclerosis (MS), neuromyelitis optica (NMO) and related NMO-spectrum disorders (NMO-SD), a pathogenic role for antibodies is primarily projected into enhancing ongoing CNS inflammation by directly binding to target antigens within the CNS. This scenario is supported at least in part, by antibodies in conjunction with complement activation in the majority of MS lesions and by deposition of anti-aquaporin-4 (AQP-4) antibodies in areas of astrocyte loss in patients with classical NMO. A currently emerging subgroup of AQP-4 negative NMO-SD patients expresses antibodies against myelin oligodendrocyte glycoprotein (MOG), again suggestive of their direct binding to CNS myelin. However, both known entities of anti-CNS antibodies, anti-AQP-4- as well as anti-MOG antibodies, are predominantly found in the serum, which raises the questions why and how a humoral response against CNS antigens is raised in the periphery, and in a related manner, what pathogenic role these antibodies may exert outside the CNS. In this regard, recent experimental and clinical evidence suggests that peripheral CNS-specific antibodies may indirectly activate peripheral CNS-autoreactive T cells by opsonization of otherwise unrecognized traces of CNS antigen in peripheral compartments, presumably drained from the CNS by its newly recognized lymphatic system. In this review, we will summarize all currently available data on both possible roles of antibodies in CNS demyelinating disorders, first, directly enhancing damage within the CNS, and second, promoting a peripheral immune response against the CNS. By elaborating on the latter scenario, we will develop the hypothesis that peripheral CNS-recognizing antibodies may have a powerful role in initiating acute flares of CNS demyelinating disease and that these humoral responses may represent a therapeutic target in its own right.

## 1. Introduction

**Figure 1:**
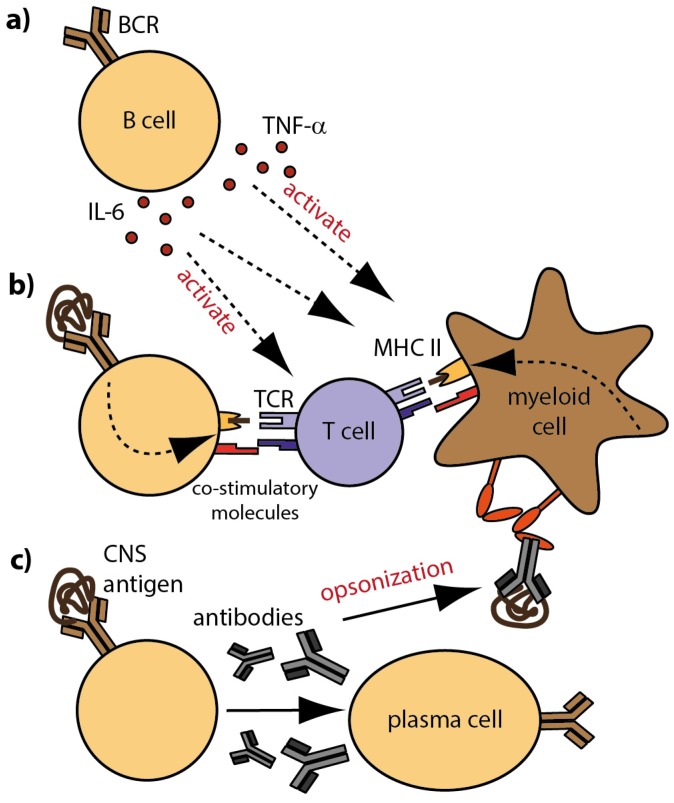
Cellular and molecular B cell properties in MS; (**a**) B cells modulate the activation and differentiation of immune cells by secretion of pro- and anti-inflammatory cytokines; (**b**) Antigen-specific B cells recognize CNS antigen via their BCR and internalize, process and present linearized antigens to responding T cells. Ligation of co-stimulatory molecules and secretion of pro-inflammatory cytokines foster the generation of effector T cells; (**c**) B cells differentiate into antibody-producing plasma cells. Secreted CNS-reactive antibodies that reach the CNS contribute to demyelination and inflammation by complement-mediated cytotoxicity. In the periphery, opsonization of rare CNS antigen by antibodies fosters the generation of auto-reactive T cells; Fc receptors on myeloid APC recognize antibody-antigen complexes and trigger internalization, processing and presentation of opsonized antigen to responding T cells. Definitions: APC = antigen-presenting cells; BCR = B cell receptor; CNS = central nervous system.

Several recent investigations highlight that B cells and antibodies can be crucially involved in the pathogenesis of central nervous system (CNS) demyelinating disorders, such as multiple sclerosis (MS), neuromyelitis optica (NMO) and NMO-spectrum disorders (NMO-SD) [1,2]. In particular the empirical success of clinical trials testing B cell-depleting anti-CD20 antibodies as therapeutic approach in MS and NMO substantiate this notion [3,4,5,6]. In these conditions, B cells are assumed to equally contribute to the inflammatory process by providing pro-inflammatory cytokines [7] and by acting as professional antigen-presenting cells (APC) [8], leading to the activation and propagation of autoreactive T cells (Figure 1). In contrast to these cellular B cell functions, the pathomechanistic involvement of antibodies may substantially differ in MS, NMO and NMO-SD.

Due to some clinical, radiological and histopathological similarities, NMO was for decades considered to be a variant of MS. The discovery of antibodies against aquaporin-4 (AQP-4), a water channel expressed on astrocytes demonstrated in an impressive manner that it is a disease in its own right [9]. The presence of these autoantibodies in the serum of patients with CNS demyelination applies now as a unique feature separating NMO from MS [10]. Although initially introduced as a diagnostic marker, more recent investigations emphasize that anti-AQP-4 antibodies are critically involved in NMO pathogenesis [11,12]. In our current understanding, classical NMO is an autoimmune astrocytopathy, where AQP-4-directed antibodies directly destroy astrocytes and demyelination occurs only as a consequence of astrocyte loss [13]. It is important to note that in NMO patients, autoantibodies are mainly detectable in the serum, but not in the cerebrospinal fluid [14,15] suggesting that NMO is a peripheral humoral autoimmune disorder. In MS in contrast, no distinct humoral immune response could be identified so far unequivocally in the periphery, but most patients present oligoclonal immunoglobulins (Ig) termed oligoclonal bands (OCB) in the cerebrospinal fluid (CSF) [16], which were mostly absent in NMO patients [17]. These OCB originate from locally supported plasma cells [18,19]. Although it is still elusive whether intrathecal Ig are pathogenic or not, they are of important diagnostic value. In addition to OCB, in a subgroup of MS patients antibody depositions are found to co-localize with complement accumulation in areas of ongoing CNS demyelination [20,21], while astrocytes remain preserved. These findings indicate that in MS lesions, myelin and/or oligodendrocytes may be directly affected.

Based on the histopathology of MS and NMO, the role of CNS-reactive antibodies was primarily projected into enhancing ongoing CNS destruction during acute disease flares [22]; at that time, the blood-brain barrier is compromised due to immune cell infiltration, and peripheral antibodies have access to the CNS. However, novel findings suggest that CNS-directed autoantibodies may be of significance even before they enter the CNS. In rodents, antibodies directed against the myelin component, myelin oligodendrocyte glycoprotein (MOG), are capable of opsonizing antigen [23] in the periphery and thereby trigger its uptake, presentation and subsequent activation of encephalitogenic T cells, resulting in experimental autoimmune encephalomyelitis (EAE) [24], an animal model for CNS demyelinating disorders.

Deciphering the pathogenic function of such autoantibodies may be of particular interest and best to study in a recently emerging group of patients with CNS demyelinating disorder in which antibodies against MOG can be detected in the serum. MOG is an extracellular component of the myelin sheath, which is exclusively expressed in the CNS and assumed to be a prime candidate autoantigen in CNS demyelinating disorders. In this context, the question arises how a peripheral immune response can be raised against antigens that are exclusively present in the CNS. In the following sections, we will summarize the current knowledge about autoantibodies in CNS demyelination. Furthermore, we will discuss how a humoral immune response against CNS antigens may be raised in the periphery and by which mechanisms CNS-reactive antibodies potentially contribute to the development of CNS demyelinating disorders.

## 2. Towards a Mechanistic Understating on the Role of Autoantibodies in CNS-Demyelinating Disorders

B cells can drive inflammation on the one hand by the secretion of pro-inflammatory cytokines and by exerting antigen-presenting function on the other hand (Figure 1a,b). Especially due to the ability of B cells to bind conformational protein-antigen specifically via their B cell receptor, they are highly competent in recognizing small amounts of protein antigen [25]. Hence, B cells are very efficient APC when they share antigen recognition with T cells [26,27] and the mere co-existence of CNS-specific B and T cells in mice is sufficient to induce EAE [28,29,30]. Another approach to induce experimental demyelination in various species is immunization with CNS antigens. One well established autoantigen activating T and B cells is MOG [31]; in mice, active immunization with MOG-derived peptides leads to the development of encephalitogenic T cells without the involvement of B cells, while immunization with conformational MOG protein additionally activates B cells in a pathogenic manner [32,33]. As a consequence, B cells differentiate into plasma cells which, in an appropriate induction regime, secrete pathogenic antibodies. Those antibodies represent the soluble counterpart of the B cell receptor and may mediate similar properties in recognizing protein antigens with a low prevalence. In EAE, MOG-specific antibodies have been shown to exacerbate ongoing disease [34,35] by promoting CNS inflammation and demyelination [36,37]. It is assumed that encephalitogenic T cells compromise the integrity of the blood-brain barrier allowing peripheral autoantibodies to enter the CNS. There, CNS-reactive antibodies can mediate myelin destruction directly by fixation of the complement system [38] and/or by increasing the uptake and intracellular metabolism of myelin by macrophages [23,39]. Lyons, Ramsbottom and Cross [40], however, suggest a more fundamental role of myelin-specific antibodies for the initiation of EAE in mice than just enhancing demyelination. They demonstrate that adoptive transfer of anti-MOG antibodies restores the ability of B cell deficient mice to develop clinical and histological EAE upon active immunization, indicating that antibodies are required for T cell (re-)activation. In the same line, studies of our group demonstrated that anti- MOG antibodies opsonize traces of otherwise undetected soluble MOG in the periphery and thereby trigger and amplify a respective pathogenic T cell response (Figure 1c). Based on this mechanism, myelin-specific antibodies in combination with myelin-reactive T cells were sufficient to induce spontaneous EAE in mice. Importantly, prior to EAE, no antibody deposition was detected within the CNS, which indicates that peripherally applied MOG-specific antibodies do not directly bind to CNS-located myelin but trigger activation of T cells outside the CNS. In conclusion, these findings indicate that peripheral CNS-specific antibodies cannot only contribute to myelin destruction by direct binding to the CNS, but additionally, promote the development of encephalitogenic T cells in the periphery.

## 3. Stratifying Human Demyelinating Disorders by the Involvement of Distinct Autoantibodies

Neuroinflammation and subsequent demyelination can be caused by a variety of extrinsic factors, but also by the immune system itself attacking endogenous molecules as it is presumably the case in MS, NMO and NMO-SD. Although the targeted antigens and pathological mechanism may differ, these diseases result in the loss of CNS myelin, neuronal damage and axonal impairment as a consequence. For decades, T cells were considered to be the major effector cell type in autoimmune CNS demyelinating disorders. However, more recent observations highlight that B cells and B cell-derived products are equally important key players in their pathogenesis. In MS, the first indication for this assumption was the discovery of OCB in the CSF of 90% of MS patients [16], which originate from locally supported plasma cells [18,19]. The particular antigen(s) recognized by these autoantibodies remain elusive and concomitantly, it is still under debate whether OCB are of pathogenic relevance for MS. However, in a subgroup of MS patients with so-called type II lesions, antibody depositions are found to co-localize with complement accumulation in areas of ongoing CNS demyelination [20,21]. This observation together with the aforementioned findings in EAE, highlight a potential role of autoantibodies for the complement-mediated destruction of myelin in acute demyelinating MS lesions [41]. Within the intrathecal humoral immune response, many potential CNS targets have been suggested in recent years, including neuroglial and astrocytic antigens such as neurofascin and contactin-2 [42,43], and also myelin antigens such as myelin basic protein and MOG [44,45], but could not be confirmed. The fact that the expression pattern of OCB in MS patients have no apparent reflection in the blood suggests that at least some of the antibodies present in the CSF are produced within the CNS. However, this comparison does not formally exclude that individual antibody entities may originate from the periphery. In line with the assumption of intrathecal IgG production, ectopic B cell follicle-like structures in the meninges of secondary progressive MS patients suggest that B cell function gradually shift from the periphery into the inflamed CNS during disease progression [46]. Furthermore, patients with primary- or secondary-progressive MS only rarely show the formation of new inflammatory spots, but rather, a gradual expansion of consisting lesions pointing towards a CNS intrinsic pathogenic mechanism that is independent of the peripheral immune system.

The heterogeneous appearance of MS, and the fact that no common autoantigen could be identified so far, suggests that MS may consist of different disease entities. Based on the discovery of anti-AQP-4 antibodies, NMO was the first condition that has been separated from the “core disorder” MS [47]. AQP-4-directed antibodies are suggested to directly destroy astrocytes [10], while demyelination occurs only as a consequence of astrocyte loss in later stages of the disease [13]. Interestingly, antibody producing plasma cells are only infrequently found in the CSF of NMO patients [48], while AQP-4 positive plasmablasts are selectively increased in the blood and shown to peak at relapses [49]. Further, OCB are only present in 15–30% of NMO patients and disappear mostly during disease progression [50]. These findings together indicate that anti-AQP-4 antibodies are mainly generated in the periphery and suggest that NMO is a peripheral humoral autoimmune disorder. However, it is poorly understood how anti-AQP-4 Ab enter the CNS and induce lesion formation. Normally, in the absence of ongoing inflammation, the CNS is assumed to be an immune-privileged organ and antibodies should not be able to cross the intact blood–brain barrier. Several observations in NMO patients support such prerequisites. Signs of viral infections prior to NMO relapses were observed in 15–30% of NMO patients, in which the virally provoked immune response may trigger an autoimmune reaction [51,52]. Further, relatively high anti-AQP-4 antibody titers can be detected in many patients during remission phase or, in individual cases, even years before disease onset, indicating the necessity of inflammatory conditions accompanying the peripheral autoantibody response [53,54]. Therefore, it is assumed that anti-AQP-4 antibodies reach the CNS only after inflammation-induced opening of the blood–brain barrier. In support of this hypothesis, disease progression occurs in NMO patients only during acute disease flares, when peripheral immune cells penetrate the CNS [55]. Apart from that, recently published in vitro data point towards the possibility that anti-AQP-4 antibodies themselves may contribute to blood–brain barrier penetration by interaction with astrocytes, which in turn prompt endothelial cells to decrease barrier function [56].

It is important to note that not all patients with a NMO-suggestive condition show anti-AQP-4 antibodies; an observation that led to the introduction of the broader category NMO-SD [57]. Within this category, a small proportion of patients show antibodies against MOG in the serum [58]. MOG is a transmembrane protein expressed on the surface of oligodendrocytes [59] and the outermost lamella of the myelin sheath. Although its exact function remains unclear, it is assumed to mediate the adhesion of neighboring myelinated fibers acting as an adhesive glue between them [60]. Due to its extracellular localization and its lack of expression in the thymus, MOG represents a plausible target for autoimmune responses [61,62]. Interestingly, patients negative for anti-AQP-4, but positive for anti-MOG antibodies fulfil many of the clinical and radiological criteria for NMO, but their relapse biology and prognosis rather resembles MS [63]. Especially the fact that the respective CNS biopsy/autopsy material showed no astrocytopathy, but demyelination as primary destructive mechanism, differentiates these patients sharply from classical NMO [64,65,66]. Consequently, these findings initiated the debate whether anti-MOG antibody-positive encephalomyelitis should be continued to be considered as a part of NMO-SD or may be defined as separate disease entity [67].

**Figure 2:**
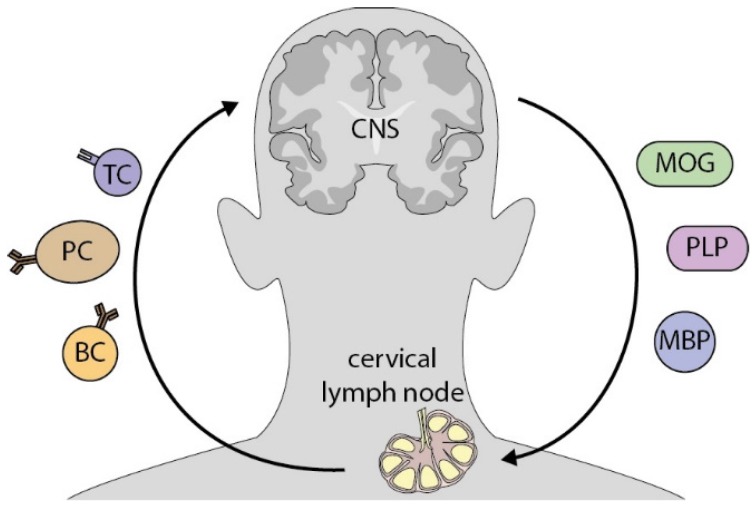
CNS antigens potentially activate peripheral immune cells in CNS-draining lymph nodes. Cerebrospinal fluid, which occasionally contains CNS antigen, such as myelin components, is drained by lymphatic vessels into deep cervical lymph nodes. There, antigen is encountered and processed by professional APC and subsequently presented to autoreactive antigen-specific T cells. By this interaction, immune cells are activated and in turn, migrate to the CNS, where they contribute to inflammation. Definitions: MOG = myelin oligodendrocyte glycoprotein; PLP = proteolipid-Protein 1; MBP = myelin basic protein; TC = T cell, PC = plasma cell; BC = B cell.

Notwithstanding these differences, the most crucial similarity between anti-MOG positive NMO-SD and anti-AQP positive NMO is that autoantibodies are predominantly found in the blood, but not in the CSF. Thus, for both conditions the question arises why and how a humoral response against CNS antigens is raised in the periphery. Furthermore, it remains elusive what pathogenic role these antibodies may exert outside the CNS and whether opsonization of endogenous CNS antigens, as described above in mice, can also occur in this human condition as a prime disease-driving mechanism. Indeed, in vitro experiments demonstrated that anti-MOG antibodies isolated from the blood of NMO-SD patients were, in principle, capable of opsonizing human MOG protein, resulting in an increased uptake of antigen by macrophages [24]. However, it remains elusive, where antibodies confer antigen recognition to myeloid APC, given that MOG is solely expressed in the CNS. A recently recognized lymphatic system which drains the CNS may represent this so far missing anatomical link between the CNS and the peripheral immune system. These newly appreciated lymphatic vessels have been shown to drain molecules from the CSF into cervical lymph nodes [68,69]. Furthermore, traces of myelin have been found there in MS patients as well as healthy controls [70,71] (Figure 2). This implies that even under non-pathologic conditions, CNS antigens can (occasionally) be transported to cervical lymph nodes. A first indication for the clinical relevance of cervical lymph has been given by a chronic-relapsing EAE model where lymphadenectomized mice show a reduced relapse severity [72].

## 4. Conclusions

B cells and antibodies have been recognized to be important players in the pathogenesis of MS, NMO and NMO-SD. While it emerges that cellular properties of B cells such as cytokine secretion and antigen presentation can drive inflammation in all of these conditions, the exact role of antibodies is still under discussion. In NMO, anti-AQP-4 antibodies are assumed to bind directly to astrocytes and induce a complement-mediated astrocytopathy. Similarly, in a subgroup of MS patients, lesions are characterized by the co-localization of antibodies and complement in areas of ongoing demyelination pointing towards antibody-mediated degradation of myelin. These mechanisms likely require, though, that the blood-brain barrier is compromised during acute disease flares and peripheral antibodies have access to the CNS, or that pathogenic antibodies are produced locally within the CNS. Notwithstanding these observations, we highlight that antibodies harbor additional pathogenic properties that may contribute to the pathogenesis of demyelinating disorders even before autoantibodies reach the CNS. Studies in mice and first transitional experiments with Ig isolated from NMO-SD patients demonstrated that CNS-reactive antibodies are capable of opsonizing soluble antigen, resulting in an increased uptake by macrophages. Subsequently, T cells differentiated in an encephalitogenic manner and induced spontaneous EAE in mice. Based on these findings, it is plausible that antibodies can contribute to the generation of auto-reactive T cells by increasing the uptake of available antigen; a mechanism probably primarily important in NMO and NMO-SD patients, where CNS-directed autoantibodies accumulate in the serum. Thus, if it can be confirmed that CNS-draining lymphoid organs are the site where antibody-mediated opsonization triggers de novo recognition of CNS antigen, both the peripheral humoral response as well as CNS draining lymph nodes may be promising targets for future therapeutic interventions in CNS demyelinating disorders. Furthermore, understanding the relative importance of antibodies for the pathogenesis of each disease entity may offer the possibility to refine treatment options in the most suitable way.
